# Expression of Plakophilins (PKP1, PKP2, and PKP3) in Gastric Cancers

**DOI:** 10.1186/1746-1596-6-1

**Published:** 2011-01-02

**Authors:** Guzin G Demirag, Yurdanur Sullu, Dilek Gurgenyatagi, Nilgun O Okumus, Idris Yucel

**Affiliations:** 1 Ondokuz Mayis University, Faculty of Medicine, Department of Medical Oncology, Samsun, Turkey; 2 Ondokuz Mayis University, Faculty of Medicine, Department of Pathology, Samsun, Turkey; 3 Ondokuz Mayis University, Faculty of Medicine, Department of Radiation Oncology, Samsun, Turkey

## Abstract

**Background:**

The importance of cell-cell junction proteins (including armadillo proteins) in tumor biology is known, but limited with regard to plakophilins. We explored the relationship between plakophilins (PKP1, PKP2, PKP3) to gastric cancer via immunohistochemical techniques.

**Methods:**

We compared the immunohistochemistry of PKPs in 34 gastric adenocarcinomas and 20 normal gastric tissues.

**Results:**

In gastric cancer, PKP1 expression was unchanged but PKP2 and PKP3 were significantly decreased as compared to normal controls. There was no observable clinical association with PKP1 or PKP2 expression; however, low PKP3 level and poor prognosis appeared to correlate with regards to node number and tumor stage. The mean disease-free survival (DFS) was 38 ± 3 months (range: 32 - 44) and mean overall survival (OS) 42 ± 4 months (range: 38 - 50). Decreased PKP2 appeared to negatively impact DFS.

**Conclusion:**

Decreased PKP2 and PKP3 may be early prognostic markers and loss of PKP3 expression during gastric carcinoma progression may indicate an invasive phenotype.

## Introduction

In the twentieth century, the highest number of cancer related deaths worldwide was attributed to gastric cancer, a disease with a dismal course and outcome [1]. Alterations of intercellular adhesion are associated with tumor dedifferentiation and invasion [2]. Armadillo-related proteins regulate functions like cell-cell interaction and cytoskeleton maintenance by producing and transducing signals influencing gene expression [3]. p120 protein is an important constituent of intercellular connections and a substrate of Src tyrosine kinase proteins. The plakophilins (PKP1, PKP2, and PKP3) are members of the armadillo-like protein subfamily, which are related to p120 protein [4]. Cell differentiation is significantly associated with the appearance of PKPs in desmosomes [5,6]. However, in all desmosome-producing cell types, only PKP2 has been found [7]. PKPs are fundamental for desmosome configuration and function [8,9]. However, little is known about the biological significance of armadillo proteins in tumorigenesis and tumor progression. In head and neck squamous cell carcinoma, PKP1 mRNA is significantly overexpressed [9]. One study reported PKP2 as expressed in all adenocarcinomas [10]. In our study, we examined PKP1, PKP2, and PKP3 expression and their clinicopathological correlation with gastric cancer.

## Materials and methods

Thirty-four gastric adenocarcinoma samples and 20 normal gastric tissue controls (patients with chronic gastritis on endoscopy) were included. Study design was approved by our institutional ethics committee and informed written consent was obtained from all patients. Hematoxylin-eosin sections were re-examined and reclassified according to World Health Organization (WHO) 2000 guidelines and the TNM staging system. Tumors were grouped into two histologic types: diffuse and intestinal. Additionally, depth of invasion (T), lymph node metastasis (N), and level of lymphatic, vascular, and perineural invasion were noted. Blocks of tumors without areas of necrosis were immunohistochemically studied for PKP1, PKP2, and PKP3. PKP immunohistochemistry was compared with stage, pathologic characteristics, and survival.

### Immunohistochemistry

All tissues were routinely fixed using 10% neutral formalin for 24 hours and subsequently embedded in paraffin. 4 μm-thick tissue sections from blocks were stained for PKP1 (mouse monoclonal, IgG1, clone PP1; Progen, Heidelberg, Germany), PKP2 (mouse monoclonal multi-epitope cocktail, IgG1, clone PP2762, PP2/86, PP2/150; Progen, Heidelberg, Germany), and PKP3 (mouse monoclonal, IgG2b, clone 23E/4; Zymed, San Francisco, CA, USA) using the streptavidin-biotin immunohistochemical technique. PKP1 and PKP2 antibodies were ready for immediate use. PKP3 was diluted to 1/100 after several dilutions were tested. Sections were deparaffinized and endogenous peroxidase activity was blocked using 3% hydrogen peroxide for 10 minutes. For antigen retrieval, sections were microwaved in 10 mM sodium citrate buffer (pH 6.0) for 15 minutes at 98°C and cooled to room temperature for 20 minutes, then rinsed in distilled water and phosphate buffered saline (PBS). A Histostain -Plus Kit (Zymed 2^nd ^generation, LAB-SA Detection System, Cat. No. 85-9643, DAB kromogen, Zymed Laboratories, San Francisco, CA, USA) was used next. After blocking solution (Reagent A) was applied for 10 minutes, PKP2 and PKP3 antibodies were applied for 1 hour at 37°C. PKP1 was applied overnight at 4°C. Sections were incubated with biotinylated anti-immunoglobulin (Reagent B) and peroxidase conjugated streptavidin (Reagent C) for 10 minutes. DAB application was performed with Reagents D1, D2, and D3 for 7 minutes according to the manufacturers' instructions. Sections were washed with phosphate buffered saline (pH 7.6) until DAB application, then washed with distilled water after DAB application. For counterstaining, Mayer's hematoxylin solution was used. As a positive control for PKP1, PKP2, and PKP3, we used skin stratified squamous epithelia. No staining was observed when PBS was used instead of the primary antibody, confirming the specifity of the primary antibody. Results were evaluated with a Leica HMLB45 (Germany, 2000) light microscope. The immunostaining results were evaluated by assigning a score based on the extent and intensity of immunoreactivity. PKP-positive cells were reported as a percentage of the total number of carcinoma cells. Based on the extent of cell labeling, the immunostaining reaction was classified as homogeneous (50% to 100%), focal (50% to 10%), or negative (0% to 10%). Homogeneous staining was scored as 6 (strong-brown), 5 (moderate-yellow), or 4 (weak-light yellow), and focal staining was scored as 3 (strong-brown), 2 (moderate-yellow), or 1 (weak-light yellow). A score of 0 indicated no staining [4].

### Statistical analysis

The non-parametric Mann-Whitney test was used to compare PKP expression between groups as the p-value did not show a normal distribution according to the Shapiro-Wilk test. Clinicopathologic characteristics and relationships were evaluated by the Chi-squared and Spearman's tests. A p-value of ≤ 0.05 was considered to be statistically significant. Survival curves were constructed using the Kaplan-Meier test.

## Results

**Figure 1:**
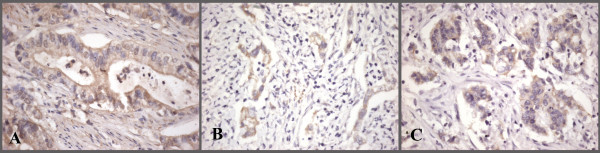
**Plakophilin immunostaining in gastric adenocarcinoma**. Focal, moderate cytoplasmic staining with PKP1(A) and PKP3(B); partial membranous staining in addition to focal, moderate cytoplasmic staining with PKP2(C) (×400).

**Figure 2:**
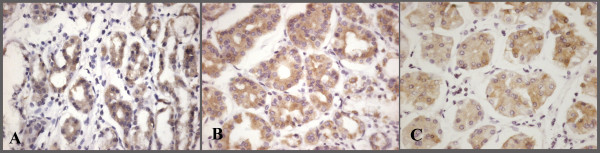
**Plakophilin immunostaining in normal gastric glands**. Homogeneous and strong cytoplasmic staining of PKP1(A), PKP2(B) and PKP3(C) (×400).

The mean age was 60.3 ± 9.7 (range 42-74) with a female to male ratio of 8/26. The mean age of controls was 59.3 ± 8.7 (range 40-73) and the female to male ratio was 5/15. There was no statistically significant difference in age and sex between groups. Tumor locations were as follows: cardia 29.4% (n = 10), fundus/body 17.6% (n = 6), antrum 38.2% (n = 13), and greater curvature 14.7% (n = 5). 13 patients (47.1%) underwent total gastrectomy and 21 patients (52.9%) subtotal gastrectomy. Pathologic characteristics were as follows: T (depth of invasion): T1 2.9% (n = 1), T2 5.9% (n = 2), T3 82.4% (n = 28), T4 8.8% (n = 3); nodal metastasis (N): N0: 5.9% (n = 2), N1 (1-6): 52.9% (n = 18), N2 (7-15): 23.5% (n = 8), N3 (> 15): 17.6% (n = 6). Stage classification was: Stage I: 2.9% (n = 1), Stage II: 2.9% (n = 1), Stage III: 70.6% (n = 24), Stage IV: 23.5% (n = 8). The following levels of invasions were noted: vascular 35.3% (n = 12), lymphatic 76.5% (n = 26), and perineural 70.6% (n = 24). 10 patients (29.%) were of the diffuse type and 24 patients (71%) were of the intestinal type (Table 1). All patients underwent postoperative chemoradiation. We observed cytoplasmic staining in 23 of 34 (68%) patients with PKP1, in 26 of 34 (76%) patients with PKP2, and in 25 of 34 (74%) patients with PKP3 (Figure 1). A few patients showed slight membranous staining in addition to cytoplasmic staining with PKP2 and PKP3 (Figure 1). In controls, strong cytoplasmic staining was observed with PKP1 in 15 of 20 (75%) patients, with PKP2 in 20 of 20 (100%) patients, and with PKP3 in 18 of 20 (90%) patients (Figure 2). A few cells stained partially membranous with PKP2 in controls. In some controls, background staining was observed with PKP1. There was no difference in PKP1 expression (p = 0.628) between groups, but PKP2 and PKP3 expression was significantly decreased in gastric cancer compared with controls (p = 0.0000001, 0.0001, respectively; Table 2).

**Table 1:** Demographic characteristics of gastric adenocarcinoma study patients.

Characteristics	n (%)
**Age (mean ± SD, range) (years)**	60.3 ± 9.7, 42-74
**Gender**	
Female	8 (24)
Male	26 (76)
**Operation**	
Total Gastrectomy	13 (47)
Subtotal Gastrectomy	21 (53)
**Localization**	
Cardia	10 (29)
Fundus/body	6 (18)
Antrum	13 (38)
Major curvatura	5 (15)
**Lauren classification**	
Intestinal	24 (71)
Diffuse	10 (29)
**Depth of invasion**	
T1	1 (3)
T2	2 (6)
T3	28 (82)
T4	3 (9)
**Lymph node metastasis**	
N0	2 (6)
N1	18 (53)
N2	8 (24)
N3	6 (18)
**Venous invasion**	12 (35)
**Lymphatic invasion**	26 (76)
**Perineural invasion**	24 (71)
**Stage**	
I	1 (3)
II	1 (3)
III	24 (71)
IV	8 (23)
**DFS **(mean ± sd, months)	38 ± 3 (32-44)
**OS **(mean ± sd, months)	42 ± 4 (38-50)
**Clinical status**	
NED	25 (74)
Death	8 (24)
With disease	1 (2)

DFS; disease-free survival; OS; Overall survival; NED, no evidence of disease

**Table 2:** Immunohistochemistry of gastric cancer and control groups.

Plakophilin expression (mean ± sd) (range)*	Gastric cancer n = 34	Controls n = 20	p-value**
PKP1	3.42 ± 2.55 (0-6)	3.60 ± 2.55 (0-6)	0.628
PKP2	2.06 ± 2.02 (0-6)	5.60 ± 0.50 (5-6)	0.0000001
PKP3	2.44 ± 2.50 (0-6)	5.00 ± 1.86 (0-6)	0.0001

*Plakophilin expression were graded according to 4 .

**Non-parametric (Mann Whitney U Test)

The staining state was separated into two parts for clinical evaluation: 0-4 and 5-6. Stages I and II were excluded from this portion of the study because they contained only one patient each. Although there was no significant correlation between PKP1 and PKP2 expression and clinical characteristics, we demonstrated a negative correlation between decreased PKP3 expression and node number and stage (p = 0.033, p = 0.007, respectively; Tables 3, 4). Local recurrences occurred in 3 patients. Eight patients developed distant metastases (bone, 3 patients; liver, 3 patients; and lung, 2 patients). The mean disease-free survival (DFS) was 38 ± 3 (range: 32 - 44) months and mean overall survival (OS) was 42 ± 4 (range: 38-50) months (long rank test). There was a significant association between decreased PKP2 expression and DFS (p = 0.02, Table 4).

**Table 3:** Correlation between PKP3 expression and nodal metastasis.

Immunohistochemistry	Lymph node status (n = 32 cases)			
	
	N0	N1	N2	N3
0-4		7	6	6
5-6	1	10	2	

Chi-squared test; p = 0.033

**Table 4:** Correlation between PKP3 expression and tumor stage.

Immunohistochemistry Staining (n = 32 cases)	Stage III	Stage IV
0-4	11	8
5-6	13	0

Chi-squared test; p = 0.007

## Discussion

Plakophilins are structural proteins and a constitutive part of the cytoplasmic plaques of desmosomes. They are engaged in the linkage of other desmosomal proteins and the recruitment of intermediate desmosomal proteins, and thus confer stability and adhesion of cells and tissues during normal development [11-13]. Recently, the clinical importance of these proteins has been studied [4,8-10,14].

PKP1 is the member of the armadillo multigene family most closely related to p120, and is thought to play a key role in stabilizing the interaction between desmosomes and the cytoskeleton in stratified squamous and complex epithelia [15]. PKP1 immunoreactivity inversely correlates with tumor grade in oropharyngeal squamous cell carcinoma (SCC) and adenocarcinoma [4,7]. In our study, however, we did not demonstrate a difference in PKP1 expression as compared to controls, or any clinical correlation for that matter.

PKP2 is present in desmosomal plaques from simple epithelium and nonepithelial tissues, and in the nucleus, whether or not desmosomes are present. This suggests that in cells lacking desmosomes, PKP2 may function exclusively in the nucleus [5,10]. Papagerakis et al. [4] showed a significant relationship between strong PKP2 immunoreactivity and metastasis in oropharyngeal SCC. In our cohort, we not only showed significantly decreased PKP2 immunoreactivity in gastric cancer tissue, but also found a significant correlation between decreased expression and a shortened DFS.

PKP3 is also present in desmosomes from simple and stratified epithelia [10]. Papagerakis et al. [4] stated that decreased PKP3 activity is inversely correlated with histological grade in oropharyngeal SCC. Reduced immunoreactivity (0-4) for PKP3 was observed primarily in poorly differentiated grade III tumors and correlated with poor survival. In contrast, Furukawa et al. [14] stated that PKP3 staining intensity in lung adenocarcinoma correlated with poor survival. PKP3 expression was significantly associated with nodal status and tumor size. Schwarz et al. [7] observed stronger PKP3 immunoreactivity in pancreatic adenocarcinoma tissue compared with normal pancreatic tissue. In our study on gastric adenocarcinoma, loss of PKP3 expression inversely correlated with nodal status and stage. Decreased PKP2 and PKP3 expression may be an early step in tumor transformation. Decreased PKP3 expression results in decreased desmosome assembly, and increased cell motility and invasion. These data lead us to propose that loss of PKP3 expression during gastric carcinoma progression may indicate an invasive phenotype. In light of these data, we suggest that during gastric carcinoma progression, the loss of PKP2 and PKP3 expression correlates with an invasive phenotype.

Initially, PKPs were identified as desmosomal plaque proteins. Subsequent studies revealed that PKPs are cytoplasmic and nuclear proteins translocated to desmosomes only during certain stages of differentiation [5,6]. Papagerakis et al. [4] detected membranous and cytoplasmic staining for PKP1, PKP2, PKP3, and PKP4 in oropharyngeal SCC. In their study, a cytoplasmic location was associated with distant metastases in PKP2, local failure in PKP1, and poor clinical outcome in PKP3. Besides abundant desmosomal staining for both PKP2 and PKP3 with apical polarity, Schwarz et al. [7] observed cytoplasmic staining for PKP3 in some adenocarcinomas. They confirmed the existence of cytoplasmic PKP3 by Western blot analysis. We observed cytoplasmic staining for PKP1, PKP2, and PKP3 in gastric carcinoma. To explain the significance of PKP location in various tumors, studies with larger numbers of patients with different tumors are required.

In summary, present study has identified the significantly decreased PKP2 and PKP3 immunoreactivity in gastric adenocarcinoma. The loss of PKP3 expression was found to be correlated with poor prognistic clinicopathological features that increased node number and advance stage of tumor. In addition, decreased PKP2 expression was associated with shorter DFS. The results of this study suggest that loss of PKP2 and PKP3 proteins may have significant role in the prognosis of gastric adenocarcinoma, however further invastigation including larger case series is needed.

## Competing interests

The authors declare that they have no competing interests.

## Authors' contributions

GGD and YS participated in study conception and design. GGD, YS, DG, NOO, and IY drafted the initial version of the manuscript and participated in data collection and analysis. GGD, YS, and IY performed data analysis and edited the manuscript. All authors read and approved the final version of the manuscript.
